# Simulating the impact on health of internalising the cost of carbon in food prices combined with a tax on sugar-sweetened beverages

**DOI:** 10.1186/s12889-016-2723-8

**Published:** 2016-02-03

**Authors:** Adam D. M. Briggs, Ariane Kehlbacher, Richard Tiffin, Peter Scarborough

**Affiliations:** 1 British Heart Foundation Centre on Population Approaches for Non-Communicable Disease Prevention, Nuffield Department of Population Health, University of Oxford, Old Road Campus, Headington, Oxford, OX3 7LF UK; 2 School of Agriculture, Policy and Development, University of Reading, Reading, RG6 6AR UK; 3 Centre for Food Security, School of Agriculture, Policy and Development, University of Reading, Reading, RG6 6AR UK

**Keywords:** Climate change, Sustainability, Social cost of carbon, Diet, Non-communicable disease, Tax

## Abstract

**Background:**

Rising greenhouse gas emissions (GHGEs) have implications for health and up to 30 % of emissions globally are thought to arise from agriculture. Synergies exist between diets low in GHGEs and health however some foods have the opposite relationship, such as sugar production being a relatively low source of GHGEs. In order to address this and to further characterise a healthy sustainable diet, we model the effect on UK non-communicable disease mortality and GHGEs of internalising the social cost of carbon into the price of food alongside a 20 % tax on sugar sweetened beverages (SSBs).

**Methods:**

Developing previously published work, we simulate four tax scenarios: (A) a GHGEs tax of £2.86/tonne of CO2 equivalents (tCO2e)/100 g product on all products with emissions greater than the mean across all food groups (0.36 kgCO2e/100 g); (B) scenario A but with subsidies on foods with emissions lower than 0.36 kgCO2e/100 g such that the effect is revenue neutral; (C) scenario A but with a 20 % sales tax on SSBs; (D) scenario B but with a 20 % sales tax on SSBs. An almost ideal demand system is used to estimate price elasticities and a comparative risk assessment model is used to estimate changes to non-communicable disease mortality.

**Results:**

We estimate that scenario A would lead to 300 deaths delayed or averted, 18,900 ktCO2e fewer GHGEs, and £3.0 billion tax revenue; scenario B, 90 deaths delayed or averted and 17,100 ktCO2e fewer GHGEs; scenario C, 1,200 deaths delayed or averted, 18,500 ktCO2e fewer GHGEs, and £3.4 billion revenue; and scenario D, 2,000 deaths delayed or averted and 16,500 ktCO2e fewer GHGEs. Deaths averted are mainly due to increased fibre and reduced fat consumption; a SSB tax reduces SSB and sugar consumption.

**Conclusions:**

Incorporating the social cost of carbon into the price of food has the potential to improve health, reduce GHGEs, and raise revenue. The simple addition of a tax on SSBs can mitigate negative health consequences arising from sugar being low in GHGEs. Further conflicts remain, including increased consumption of unhealthy foods such as cakes and nutrients such as salt.

**Electronic supplementary material:**

The online version of this article (doi:10.1186/s12889-016-2723-8) contains supplementary material, which is available to authorized users.

## Background

Rising global temperatures as a consequence of climate change are likely to have major implications for human health [1]. Up to 30 % of global greenhouse gas emissions (GHGEs) are estimated to arise from agriculture and associated land use change [2]. In the UK, the figure is around 10 % of total emissions (not including land-use change) [3]. Changing food consumption patterns therefore offer an important potential contribution to the overall UK target of an 80 % reduction in GHGEs from 1990 levels by 2050 [4, 5].

The majority of previous observational and modelling studies have identified synergies between diets that are low in GHGEs and beneficial for health; for example red meat is very high in GHGEs and also has detrimental health consequences when consumed [6–15]. Although broadly a consistent finding, a diet that is healthy for the planet may not necessarily be better for individuals’ health. Studies of French dietary survey data concluded that foods and diets of high nutritional quality have higher GHGEs than those of low nutritional quality [16–18], and Briggs et al. found that some food and drink, such as sugar and sugar-sweetened beverages (SSBs), are both very low in GHGEs and bad for health [7]. Furthermore, a study by Biesbroek et al. found that among the EPIC-NL cohort there is no correlation between mortality and dietary derived GHGEs [19].

There is a potential market failure in agriculture as the true social cost of carbon (the wider costs to society of GHGEs, such as their direct and indirect impacts on food production and health) is not included in the price of food and therefore is neither paid for nor is visible to the consumer. Previous studies have simulated the possible implications for population diets of modifying food prices to shift consumption patterns away from foods with high levels of GHGEs [7, 13, 20]. Briggs et al. modelled the possible implications on UK population diet and health of internalising the social cost of carbon [7]. They found broad synergies between consuming foods with lower GHGEs and improving population health, however the price changes led to increased consumption of unhealthy sugar and SSBs.

In this study, we investigate using food pricing policies to shift the UK population towards a diet that is both healthier and more sustainable (has lower GHGEs). Food pricing policies are increasingly being investigated as a potential mechanism to improve population diets [21–26]. In the UK, taxes on SSBs are being increasingly debated as a possible policy option with both the parliamentary health committee and the government’s public health department suggesting that they should be used to help combat population obesity [27, 28]. Beyond this, several countries have implemented unhealthy food and SSB taxes motivated at least in part to improve health, for example Hungary, France, Denmark, and Mexico [29, 30]. More recently the UK thinktank, Chatham House, recommended that the UK government should consider a tax on meat and other unsustainable products to help tackle climate change [31]. This study aims to further characterise a healthy sustainable diet and to directly address the undesirable health consequences of SSBs being low in GHGEs. We estimate and compare the impact on non-communicable disease mortality and GHGEs in the UK of incorporating the social cost of carbon into the price of food through a tax, with and without a 20 % tax on SSBs.

## Methods

**Fig. 1:**
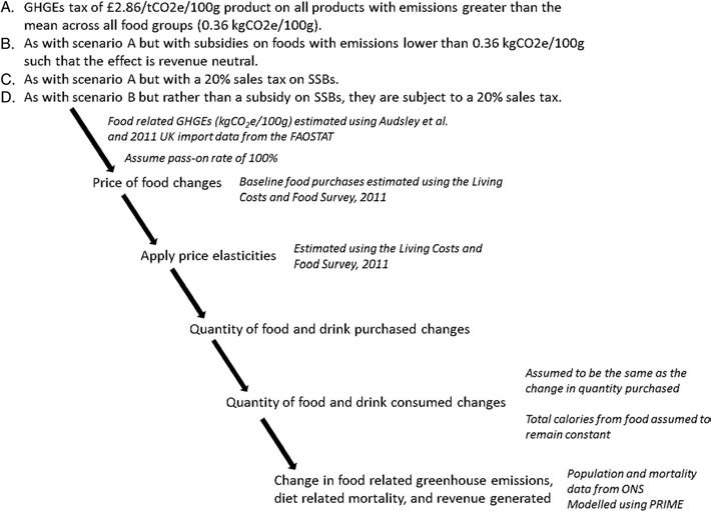
Modelling pathway. SSBs, sugar sweetened beverages; GHG, greenhouse gas; kgCO2e, kilograms of CO2 equivalents; ONS, Office for National Statistics; FAOSTAT, Food and Agriculture Organisation of the United Nations Statistical Division

We model the impact on non-communicable disease mortality and agriculture-associated GHGEs in the UK of four tax scenarios using a previously published approach with some methodological changes [7]. The modelling pathway and its major assumptions are illustrated in Fig. 1.

### Tax scenarios

Tax rates are based on Moran et al.’s agriculture marginal abate cost curve (MACC) produced for the UK Government’s Department for Environment, Food, and Rural Affairs (Defra) [32]. This estimates the cost-effectiveness of different GHG abatement strategies and plots them such that the user can visualise how many GHGEs can be saved with a given level of investment. This MACC suggests that 7850 kilotonnes of CO2 equivalents (ktCO2e) can be saved at a cost of £24.10/tCO2e (£28.61/tCO2e, 2011 prices); the next most cost effective strategy costs £206.83/tCO2e (2011 prices). The lower cost aligns with Defra’s estimate of the UK shadow price of carbon of £28 (2011 prices), based on Stern’s estimate of the social cost of carbon but taking into account the UK costs of abatement and political willingness to act [33, 34]. It should be noted that the estimate of the social cost of carbon does vary [35].

Four tax scenarios are modelled to illustrate the potential effect on non-communicable disease mortality, change in GHG emissions, and revenue generated in the UK of changing food prices to incorporate the cost of greenhouse gas emissions. Scenarios A and B repeat previous taxes simulated by Briggs et al. [7], and scenarios C and D combine scenarios A and B with a SSB tax. Scenarios A and B are repeated from previous work to allow for direct comparisons to be made between taxes with and without the SSB tax using comparable methods and updated data sources [7]. A SSB is defined as a soft drink with added sugar, comprising of both concentrated and non-concentrated beverages and including energy drinks and fruit juice with added sugar. This group does not include alcoholic drinks or drinks served hot. The scenarios are as follows:

A. GHGEs tax of £2.86/tCO2e/100 g product on all products with emissions greater than the mean across all food groups (0.36 kgCO2e/100 g).
B. As with scenario A but with subsidies on foods with emissions lower than 0.36 kgCO2e/100 g such that the effect is revenue neutral.
C. As with scenario A but with a 20 % sales tax on SSBs.
D. As with scenario B but with a 20 % sales tax on SSBs.

Levels of subsidy in scenario B are derived based on a revenue neutral outcome before the implementation of the tax and therefore before any change in purchasing patterns. A 20 % SSB sales tax is chosen based on current evidence and opinion that a tax of at least 20 % tax is required for meaningful population health benefits [21, 26, 36].

### Food and GHG emissions

Baseline consumption data are taken from the Living Costs and Food Survey (LCF), 2011 [37]. This is a representative purchasing survey of 256 food categories using two-week food expenditure diaries of 5,531 households.

GHG emissions are derived using methods described previously [7]. In summary, GHG emissions data are taken from Audsley et al. who assembled a near complete list of emissions per kg of food product consumed in the UK, but produced in three geographical regions: the UK, elsewhere in Europe, or elsewhere in the world [38]. Emissions per 100 g of each food product are based on emissions from land use change (LUC, those emissions resulting from altering land for agriculture, for example converting forest into pasture) and primary production up to the retail distribution centre (pre-RDC, therefore not including distribution to retail units, cooking, and waste disposal). In order to derive emissions per 100 g product for each food category in the LCF, UN FAOSTAT was used to identify the proportion of each product produced domestically, imported from Europe, or imported from elsewhere in the world [39]. Food waste is assumed not to change following the implementation of tax. The mean level of emissions is a UK aggregate level consumption-weighted average calculated from UN FAOSTAT data.

### Deriving and applying price elasticities

Price elasticities measure how purchases change with a 1 % change in price. Own price elasticities measure how demand for a good is affected bya change in its price; and cross-price elasticities measure the response to changes in the prices of other goods. For example, if milk has an own-price elasticity of −0.8, a 10 % increase in the price of milk will result in an 8 % reduction in milk purchases. If the cross price elasticity of milk and cereal is −0.2, a 10 % increase in the price of milk will result in a 2 % reduction in the amount of cereal purchased and cereal is said to be a complement to milk. Vice versa, a positive cross price elasticity indicates a substitute relationship. The inclusion of cross price elasticities in our analysis is critical in enabling us to represent the impacts of a tax by reflecting the patterns of substitution that accompany the changes in the products that are specifically targeted.

**Fig. 2:**
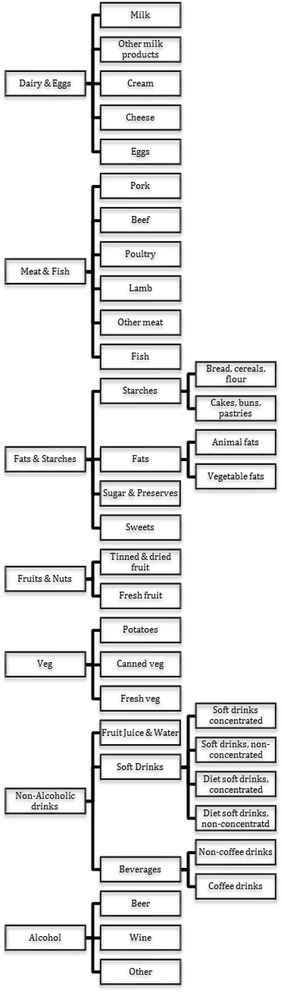
Categorisation of food and drinks in hierarchical Almost Ideal Demand System

Using the LCF 2011, 256 food-categories were allocated to one of 32 different food groups each sharing related food and drink products (Fig. 2). A Quadratic Almost Ideal Demand System was estimated using methods based on those described in Tiffin and Arnoult [40] and employed by Briggs et al. [7]. The model was estimated as a hierarchy of sub-models with a top-level model which has seven food groups (dairy and eggs, meat and fish, fats and starches, fruits and nuts, vegetables, non-alcoholic drinks, alcohol). Next, seven subsystems were estimated which represent the household's decision to allocate its respective food group budgets between disaggregated food groups. Finally, four subsystems are estimated for starches, fats, soft drinks (divided into four separate categories rather than the one used previously by Briggs et al. [7]), and alcoholic beverages. SSBs are comprised of two categories of the Living Costs and Food Survey (LCF), 2011:“soft drinks, concentrated, not low calorie” and “soft drinks, not concentrated, not low calorie”. All conditional elasticities which constitute the mean of all household specific elasticity matrices were then combined following Edgerton (1997) to obtain unconditional elasticities over all stages of the model in which category expenditure is free to vary (see Additional file 1 for complete price elasticity matrix) [41].

Based on the tax rate, the resultant price changes were computed and then applied to each food group for each scenario (assuming that the tax is fully passed on to the consumer). By summing over all own- and cross-price effects we computed changes in household food purchases.

Table 1 shows the GHG emissions associated with each food group and the tax applied for each of the scenarios A, B, C, and D.

**Table 1:** Greenhouse gas emissions per kg for each food category and the price changes simulated under each tax scenario

Food group	GHGe/kg product (kgCO2e)	Tax/kg product for each scenario (£)			
		A	B	C	D
Milk	1.8	0	−0.05	0	−0.05
Other milk products	2.4	0	−0.04	0	−0.04
Cream	2.4	0	−0.04	0	−0.04
Cheese	1.8	0	−0.05	0	−0.05
Eggs	4.9	0.03	0.04	0.04	0.04
Pork	7.9	0.12	0.12	0.12	0.12
Beef	66.1	1.79	1.79	1.79	1.79
Poultry	5.4	0.05	0.05	0.05	0.05
Lamb	64.3	1.74	1.74	1.74	1.74
Other meat	38.9	1.01	1.01	1.01	1.01
Fish	5.4	0.05	0.05	0.05	0.05
Bread, cereals, flour	0.8	0	−0.08	0	−0.08
Cakes, buns, pastries, biscuits	1.5	0	−0.06	0	−0.06
Animal fats	35.8	0.92	0.92	0.92	0.92
Vegetable fats	2.8	0	−0.02	0	−0.02
Sugar and preserves	0.1	0	−0.10	0	−0.10
Sweets	0.1	0	−0.10	0	−0.10
Tinned and dried fruit and nuts	0.8	0	−0.08	0	−0.08
Fresh fruit	0.8	0	−0.08	0	−0.08
Potatoes	0.4	0	−0.09	0	−0.09
Canned veg	1.0	0	−0.08	0	−0.08
Fresh veg	1.0	0	−0.08	0	−0.08
Fruit juice	0.8	0	−0.08	0	−0.08
Soft drinks, concentrated, low calorie	0.1	0	−0.10	0	−0.10
Soft drinks, not concentrated, low calorie	0.1	0	−0.10	0	−0.10
Soft drinks, concentrated, not low calorie	0.1	0	−0.10	0.004 (20 %)	0.004 (20 %)
Soft drinks, not concentrated, not low calorie	0.1	0	−0.10	0.017 (20 %)	0.017 (20 %)
Non-coffee drinks	1.9	0	−0.05	0	−0.05
Coffee drinks	10.1	0.19	0.19	0.19	0.19
Beer	3.8	0.01	0.01	0.01	0.01
Wine	1.0	0	−0.07	0	−0.07
Other alcohol	3.5	0	−0.00	0	−0.00

*GHGEs* greenhouse gas emissions, *kgCO2e* kilograms of CO2 equivalents

### Calculating change in non-communicable disease mortality, GHG emissions, and revenue generated

The impact of the new diet on UK non-communicable disease mortality following each tax scenario is estimated using the PRIME comparative risk assessment model (previously known as DIETRON) [7, 42]. The PRIME model estimates the age- and sex-specific effect on non-communicable disease mortality of 11 dietary variables in five-year age groups from 15 years to 85+ years using relative risk estimates derived from meta-analyses. Dietary input data are total calories consumed per day, total salt, total fibre, total fruit (g), total vegetables (g), alcohol (g), and total cholesterol consumed, and percentage of energy derived from total fat, saturated fat, poly-unsaturated fat, and mono-unsaturated fat. A detailed description of the model is available elsewhere [42]. As with previous modelling and consistent with empirical research indicating that liquid calories are non-satiating, food calories are assumed to remain constant and liquid are assumed to change (liquid calories consisted of the following food groups: Milk; Fruit juice; Soft drinks, concentrated, low calorie; Soft drinks, not concentrated, low calorie; Soft drinks, concentrated, not low calorie; Soft drinks, not concentrated, not low calorie; Non-coffee drinks; Coffee drinks; Beer; Wine; Other alcohol) [36, 43–45].

All uncertainty estimates are based on 95 % credible intervals surrounding the price elasticity estimates. These are calculated using a sample of 10,000 iterations obtained using Markov Chain Monte Carlo methods. A further 2000 observations are discarded from the beginning of the sample as a burn-in. As with previous work, the uncertainty surrounding the price elasticity estimates is where the greatest uncertainty lies in the model [7, 36]. These therefore produce wider intervals than if estimated using probabilistic uncertainty analyses based on the relative risk estimates found in PRIME, and are therefore more conservative. The econometric model and the PRIME model are not linked, to estimate the uncertainty of combining both models is currently beyond our available computing power.

## Results

**Fig. 3:**
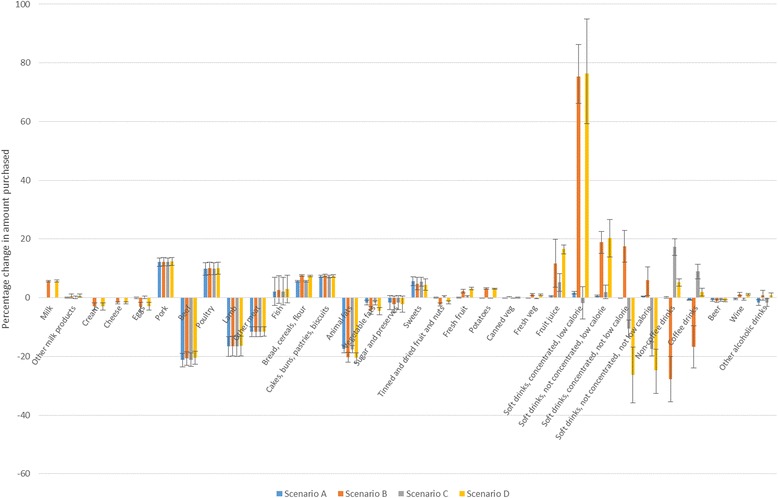
Percentage change in amount of each food group purchased following each tax scenario

Following the implementation of each tax scenario, meats and non-alcoholic beverages experienced the largest changes in the amount purchased. Across all four tax scenarios, purchases of *beef*, *lamb*, and *other meat* significantly decreased by approximately 21 %, 17 %, and 12 % respectively, alongside 12 % and 10 % increases in the amount of *pork* and *poultry* purchased respectively (Table 2, Fig. 3). There were also significant increases across all the scenarios in purchases of *bread, cereals, and flour*; *cakes, buns, pastries, and biscuits*; and *sweets*. Scenarios with subsidies (B and D) resulted in significant reductions in purchases of *cream*, *cheese*, and *eggs* (approximately 3 %, 2 %, and 3 % respectively) and increases in *fresh fruit* and *potatoes* of approximately 3 %; there were minimal changes to purchases of these food groups in scenarios A and C.

**Table 2:** Percentage change in amount of each food group purchased following each tax scenario

Food group	Percentage change in amount purchased for each scenario			
	A	B	C	D
Milk	0.01 (−0.04 to 0.05)	5.36 (5.32 to 5.94)	0.02 (−0.04 to 0.09)	5.65 (5.35 to 5.96)
Other milk products	0.03 (−0.21 to 0.27)	0.55 (−0.11 to 1.21)	0.10 (−0.27 to 0.47)	0.69 (0.18 to 1.18)
Cream	0.00 (−0.00 to 0.01)	−2.94 (−2.06 to −1.43)	0.01 (−0.01 to 0.02)	−2.94 (−4.16 to −1.75)
Cheese	0.01 (−0.04 to 0.05)	−1.75 (−2.02 to 0.05)	0.02 (−0.05 to 0.09)	−1.72 (−2.03 to −1.41)
Eggs	0.01 (−0.26 to 0.27)	−3.00 (−4.54 to −1.61)	−0.08 (−0.31 to 0.49)	−2.85 (−4.29 to −1.52)
Pork	12.10 (10.73 to 13.46)	12.24 (10.88 to 13.60)	12.13 (10.76 to 13.49)	12.30 (10.94 to 13.65)
Beef	−21.26 (−23.52 to −18.97)	−20.71 (−23.03 to −18.39)	−21.15 (−23.40 to −18.86)	−20.50 (−22.75 to −18.23)
Poultry	9.80 (7.70 to 11.86)	10.01 (7.90 to 12.09)	9.84 (7.74 to 11.90)	10.10 (8.01 to 12.17)
Lamb	−16.62 (−19.99 to −13.31)	−16.49 (−19.88 to −13.19)	−16.60 (−19.95 to −13.29)	−16.43 (−19.80 to −13.14)
Other meat	−11.60 (−13.23 to −9.94)	−11.58 (−13.20 to −9.92)	−11.60 (−13.22 to −9.93)	−11.57 (−13.19 to −9.91)
Fish	2.01 (−2.66 to 6.91)	2.66 (−1.95 to 7.47)	2.14 (−2.53 to 7.03)	2.91 (−1.67 to 7.69)
Bread, cereals, flour	5.67 (5.40 to 5.95)	7.50 (7.21 to 7.79)	5.60 (5.32 to 5.88)	7.36 (7.09 to 7.62)
Cakes, buns, pastries, biscuits	7.29 (6.88 to 7.72)	7.52 (7.06 to 7.99)	7.19 (6.78 to 7.62)	7.33 (6.90 to 7.77)
Animal fats	−17.53 (−18.78 to −16.20)	−20.31 (−21.97 to −18.53)	−17.62 (−17.86 to −16.28)	−20.47 (−22.13 to −18.71)
Vegetable fats	−1.61 (−2.41 to −0.87)	−4.37 (−5.69 to −3.08)	−1.72 (−2.51 to −0.96)	−4.56 (−5.87 to−3.29)
Sugar and preserves	−1.72 (−4.15 to 0.78)	−2.23 (−4.89 to 0.56)	−1.72 (−4.15 to 0.78)	−2.23 (−4.90 to 0.56)
Sweets	5.62 (4.14 to 7.18)	4.66 (2.72 to 6.96)	5.44 (3.95 to 6.99)	4.32 (2.47 to 6.48)
Tinned and dried fruit and nuts	0.00 (−0.20 to 0.21)	−2.29 (−2.80 to −1.73)	0.38 (0.07 to 0.70)	−1.58 (−2.06 to −1.08)
Fresh fruit	0.00 (−0.24 to 0.25)	2.25 (1.70 to 2.82)	0.45 (0.09 to 0.81)	3.12 (2.74 to 3.50)
Potatoes	−0.04 (−0.11 to 0.02)	3.15 (2.94 to 3.37)	−0.07 (−0.16 to 0.02)	3.10 (2.92 to 3.29)
Canned veg	−0.04 (−0.10 to 0.02)	0.11 (−0.06 to 0.29)	−0.07 (−0.15 to 0.02)	0.06 (−0.09 to 0.21)
Fresh veg	−0.09 (−0.22 to 0.04)	0.14 (0.81 to 1.48)	−0.14 (−0.33 to 0.03)	1.03 (0.78 to 1.29)
Fruit juice	0.55 (0.32 to 0.78)	11.71 (3.51 to 19.89)	5.17 (2.17 to 8.11)	16.54 (14.99 to 18.01)
Soft drinks, concentrated, low calorie	1.74 (1.28 to 2.22)	75.29 (66.13 to 86.23)	−1.87 (−7.21 to 3.68)	76.39 (59.34 to 94.93)
Soft drinks, not concentrated, low calorie	0.63 (0.44 to 0.82)	18.88 (15.18 to 22.51)	1.97 (−0.37 to 4.29)	20.29 (13.89 to 26.65)
Soft drinks, concentrated, not low calorie	−0.03 (−0.10 to 0.05)	17.42 (12.13 to 22.93)	−10.47 (−13.35 to −7.61)	−26.24 (−35.75 to −16.79)
Soft drinks, not concentrated, not low calorie	0.44 (0.29 to 0.58)	5.96 (0.79 to 10.44)	−17.47 (−19.93 to −15.06)	−24.80 (−32.59 to −17.60)
Non-coffee drinks	0.10 (−0.08 to 0.29)	−27.68 (−35.55 to −20.01)	17.23 (14.42 to 20.15)	5.20 (3.94 to 6.45)
Coffee drinks	−0.74 (−0.85 to −0.64)	−16.67 (−23.87 to −9.64)	8.90 (6.45 to 11.42)	1.86 (0.52 to 3.17)
Beer	−0.85 (−1.14 to −0.58)	−1.07 (−1.58 to −0.57)	−0.85 (−1.25 to −0.46)	−1.06 (−1.35 to −0.77)
Wine	−0.45 (−0.76 to −0.14)	1.23 (0.67 to 1.79)	−0.45 (−0.88 to 0.00)	1.23 (0.95 to 1.51)
Other alcohol	−1.70 (−2.64 to −0.77)	0.79 (−0.86 to 2.43)	−1.69 (−3.02 to −0.34)	0.80 (0.03 to 1.58)

95 % credible intervals in parentheses

Significant differences in the change in purchases of non-alcoholic drinks categories were found between the four tax scenarios. In scenarios B and D, where drinks low in GHGEs are subsidised, there were significant increases in purchases of *milk* (5.4 % [95 % CI 5.3 % to 5.9 %] and 5.6 % [5.4 to 6.0] increases in scenarios B and D respectively), *fruit juice* (11.7 % [3.5 to 19.9] and 16.5 % [2.7 to 3.5]), *low calorie concentrated soft drinks* (75.3 % [66.1 to 86.2] and 76.4 % [59.3 to 94.9]), and *low calorie not concentrated soft drinks* (18.9 % [15.2 to 22.5] and 20.3 % [13.9 to 26.7]). Smaller changes in the amounts purchased were found in scenarios A and C. Larger increases in purchases were seen for *low calorie concentrated soft drinks* compared to *low calorie not concentrated soft drinks* in scenarios B and D despite the same price change because the subsidy resulted in a much larger relative price change for *low calorie concentrated soft drinks* (which also has a larger price elasticity) than *not concentrated.*

Taxing SSBs in scenarios C and D resulted in significant reductions in the two *not low calorie* soft drink categories, with greater reductions found in scenario D than C (Table 2, Fig. 3). This is compared to either no change or increases in the same categories in scenarios A and B. However, given that households spend on average between 0.18 % and 0.9 % of their food expenditure on the four soft drinks categories, changes in absolute quantities are small (see Additional file 2 for expenditure shares). Also of note, there were significant changes in purchases of *coffee drinks* and *non-coffee drinks* categories in all scenarios, varying in both magnitude and direction. For example, in scenario B there were significant reductions of 27.7 % (20.0 to 35.6) and 17.7 % (9.6 to 23.9) of *coffee drinks* and *non-coffee drinks* categories respectively whereas adding a SSB tax in scenario D resulted in smaller but still significant increases in the same categories of 1.9 % (0.5 to 3.2) and 5.2 % (3.9 to 6.5).

The new purchasing patterns that arose as a consequence of the different tax scenarios had varying effects on dietary nutrient composition (Table 3). Scenario A resulted in no change to daily energy intake (and no significant changes to kcal/day derived from individual drinks categories) however in scenario B, total daily kcal consumed increased by 13 kcal/day (95 % CI: 8 to 18). The increase in total kcal/day was from increases in consumption of milk (6.9 kcal [6.5 to 7.3]), fruit juice (2.0 kcal [0.6 to 3.3]), concentrated soft drinks (2.6 kcal [1.8 to 3.4]), and non-concentrated soft drinks (1.8 kcal [0.2 to 3.2]). The magnitude of the increase was offset in part by a reduction in energy intake from coffee and non-coffee drinks (1.4 kcal [0.9 to 1.8) and much smaller changes to other drinks categories. Adding a tax on SSBs in scenario C led to a reduction from baseline total daily energy intake by 5 kcal/day (3 to 8) due to less concentrated soft drink consumption (1.5 kcal [1.1 to 2.0]) and non-concentrated soft drink consumption (5.3 kcal [4.5 to 6.0) alongside small increases in coffee and non-coffee drink consumption. The SSB tax in scenario D resulted in no significant change to daily energy intake. Energy intakes from milk and fruit juice increased by the similar amounts to scenario B (6.9 kcal [6.6 to 7.3] and 2.8 kcal [2.5 to 3.0] respectively) but large falls in energy intake from concentrated (3.9 kcal [2.5 to 5.3]) and non-concentrated SSBs (7.5 kcal [5.3 to 9.8]) meant that overall daily kcal intake remained unchanged.

**Table 3:** Nutrient composition of baseline and simulated scenarios

	Nutrient composition of baseline diets and under each scenario (calories from drinks only allowed to change)				
	Baseline	A	B	C	D
Energy (kcal/day)	2000	2000 (2000 to 2000)	2013 (2008 to 2018)	1995 (1992 to 1997)	1999 (1995 to 2004)
Total fat (% total energy)	37.0	36.3 (36.3 to 36.4)	35.9 (35.9 to 35.9)	36.5 (36.4 to 36.5)	36.2 (36.2 to 36.2)
SAFAs (% total energy)	14.4	14.1 (14.1 to 14.1)	13.9 (13.9 to 13.9)	14.1 (14.1 to 14.1)	14.0 (14.0 to 14.0)
MUFAs (% total energy)	13.6	13.4 (13.4 to 13.4)	13.2 (13.2 to 13.2)	13.4 (13.4 to 13.4)	13.3 (13.3 to 13.3)
PUFAs (% total energy)	6.6	6.5 (6.5 to 6.5)	6.4 (6.4 to 6.4)	6.5 (6.5 to 6.5)	6.4 (6.4 to 6.4)
Cholesterol (mg/day)	228.2	223.0 (221.9 to 224.0)	221.2 (219.6 to 222.7)	223.1 (222.0 to 224.2)	221.4 (219.9 to 222.9)
Fibre (g/day)	13.2	13.3 (13.4 to 13.3)	13.5 (13.5 to 13.4)	13.3 (13.4 to 13.3)	13.5 (13.5 to 13.4)
Salt (mg/day)	6196	6221 (6222 to 6221)	6247 (6247 to 6246)	6221 (6220 to 6222)	6244 (6244 to 6243)
Fruit and vegetables (g/day)	344.3	339.9 (341.6 to 338.1)	346.9 (345.3 to 348.4)	342.2 (342.7 to 341.7)	349.7 (351.0 to 348.4)
Sugar (g/day)	113.7	113.9 (113.8 to 114.0	115.9 (114.8 to 117.0)	112.4 (111.9 to 112.9)	112.2 (111.1 to 113.4)
Iron (mg/d)	10.6	10.6 (10.6 to 10.6)	10.7 (10.6 to 10.7)	10.7 (10.7 to 10.6)	10.7 (10.7 to 10.7)
Calcium (mg/d)	883.7	890.2 (891.7 to 888.6)	907.2 (906.9 to 907.6)	890.3 (891.3 to 889.4)	907.3 (907.4 to 907.2)
Zinc (mg/d)	8.2	8.0 (8.0 to 8.0)	8.1 (8.1 to 8.1)	8.1 (8.0 to 8.1)	8.1 (8.1 to 8.1)
Vitamin A (μg /day)	794.5	756.1 (756.3 to 756.0)	754.7 (753.3 to 756.1)	755.4 (755.2 to 755.7)	752.1 (750.6 to 753.6)
Vitamin D (μg /day)	2.6	2.6 (2.6 to 2.6)	2.6 (2.6 to 2.6)	2.6 (2.6 to 2.6)	2.6 (2.6 to 2.6)
Vitamin B12 (μg /day)	5.7	5.5 (5.5 to 5.6)	5.6 (5.5 to 5.7)	5.5 (5.5 to 5.6)	5.6 (5.6 to 5.7)

95 % credible intervals in parentheses. SAFAs, saturated fatty acids; MUFAs, mono-unsaturated fatty acids; PUFAs, poly-unsaturated fatty acids

All tax scenarios resulted in significant reductions in total fat (% of total energy), saturated fatty acids (% of total energy) and cholesterol (mg/day) intake, and significant increases in fibre. Fruit and vegetable consumption varied between scenarios with significant increases in consumption in scenarios B and D, where fruit and vegetables are subsidised, and significant reductions in scenarios A and C. Sugar consumption significantly increased in both scenarios A and B but the introduction of the SSB tax in scenarios C and D resulted in total sugar consumption being significantly reduced.

There were small changes in micronutrient composition of the diet across all scenarios with small statistically significant reductions in zinc, vitamin A, and vitamin B12 consumed (although they remain above recommended levels) [46] alongside a significant increase in calcium consumed.

**Fig. 4:**
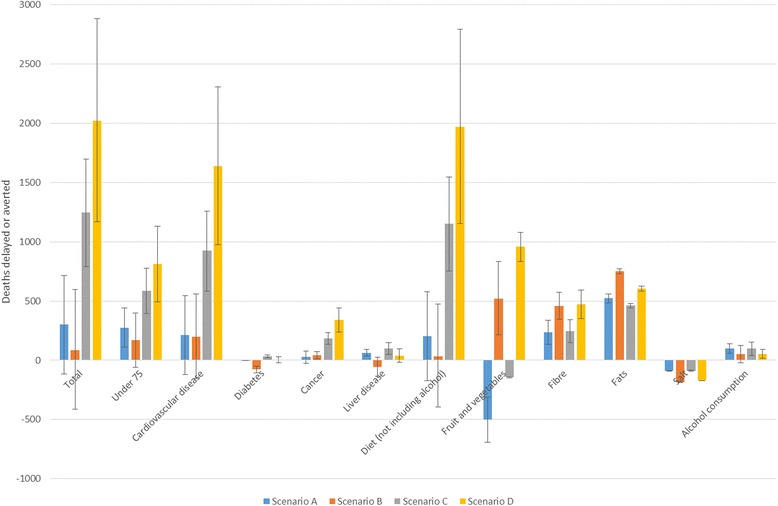
Deaths delayed or averted following each tax scenario

All scenarios delay or avert deaths, although results were non-significant in scenarios A and B (Table 4, Fig. 4). Across all scenarios, the majority of deaths delayed or averted can be attributed to increases in fibre intake and reductions in fat consumption; an increase in salt consumption resulted in significant changes in the other direction. Increases in fruit and vegetable consumption in scenarios B and D (where fruit and vegetables are subsidised) resulted in significant numbers of deaths delayed or averted. This is compared to significant reductions in fruit and veg in scenarios A and C. Health outcomes are predominantly attributable to changes in deaths from cardiovascular disease and cancer.

**Table 4:** Deaths delayed or averted attributable to different foods, nutrients, and diseases

	Deaths delayed or averted under each scenario			
	A	B	C	D
Total	302 (−116 to 718)	86 (−412 to 599)	1249 (792 to 1699)	2023 (1169 to 2884)
Total (<75 years)	276 (112 to 439)	169 (−58 to 401)	586 (395 to 775)	811 (493 to 1131)
Diet (excluding obesity)	171 (−156 to 497)	1545 (1367 to 1721)	477 (349 to 598)	1861 (1597 to 2117)
Diet (including obesity)	204 (−174 to 580)	33 (−393 to 473)	1152 (752 to 1545)	1970 (1154 to 2794)
Fruit and vegetables	−500 (−692 to −311)	521 (209 to 829)	−142 (−149 to −142)	960 (835 to 1077)
Fibre	237 (136 to 339)	459 (345 to 573)	245 (147 to 342)	472 (351 to 592)
Fats	522 (485 to 560)	750 (729 to 771)	461 (442 to 481)	605 (582 to 623)
Salt	−90 (−91 to −89)	−183 (−184 to −180)	−88 (−93 to −84)	−171 (−170 to −169)
Alcohol consumption	98 (58 to 138)	53 (−19 to 125)	97 (40 to 154)	52 (14 to 90)
Cardiovascular disease	213 (−121 to 546)	198 (−154 to 559)	925 (584 to 1259)	1638 (975 to 2306)
Diabetes	−1 (−2 to 1)	−77 (−106 to −48)	31 (19 to 44)	4 (−23 to 32)
Cancer	26 (−27 to 79)	40 (10 to 72)	185 (135 to 234)	340 (239 to 442)
Liver disease	62 (35 to 91)	−55 (−136 to 26)	99 (48 to 150)	39 (−17 to 95)

95 % credible intervals in parentheses

Each scenario led to statistically significant reductions in GHGEs of between 16.5 million tonnes CO2e (12.7 to 20.1) in scenario D (smallest) and 18.9 million tonnes CO2e (15.4 to 22.3) in scenario A (largest, Table 5). Reductions were similar across all scenarios (credible intervals overlap), and the majority of the reduction was attributable to LUC, accounting for 84–90 % of all reduced emissions.

**Table 5:** Changes to greenhouse gas emissions and the potential revenue generated under each tax scenario

	Change in GHGEs and revenue generated in each scenario			
	A	B	C	D
Reduction in total emissions (ktCO2e)	18,866 (15,367 to 22,338)	17,082 (13,174 to 20,991)	18,537 (14,947 to 22,094)	16,453 (12,776 to 20,108)
Reduction in LUC emissions (ktCO2e)	15,809 (13,512 to 18,092)	15,070 (12,635 to 17,512)	15,677 (13,362 to 17,974)	14,819 (12,459 to 17,170)
Revenue generated (£, 000 s)	3,036 (2,956 to 3,117)	−537 (−603 to −472)	3,418 (3,328 to 3,509)	−124 (−202 to −46)

95 % credible intervals in parentheses. GHGEs, greenhouse gas emissions; ktCO2e, kilotonne of carbon dioxide equivalents

Scenarios A and C (non-subsidised) have the possibility of generating £3.0 billion and £3.4 billion in revenue respectively, with the difference being attributable to the 20 % tax on SSBs in scenario C. Scenario B is designed to be revenue neutral, however following the changes in purchasing as a result of the tax, there would be a £540 m reduction in revenue as people move away from taxed food products. In scenario D where SSBs are also taxed, the result is a £120 m reduction in revenue.

## Discussion

This study gives further clarification of the synergies and tensions between diets that are low in GHGEs and their potential effects on population health. A problem of changing food prices based exclusively on GHGEs is that some food products, such as sugar, are low in GHGEs. This may mean that these products are consumed more, leading to negative health outcomes. Our results suggest that the addition of a 20 % tax on SSBs to a food tax based on GHGEs could mitigate the negative health consequences of increased sugar consumption whilst still significantly reducing GHGEs. We find that combining a food-based GHGE tax with a tax on SSBs has the potential to reduce UK food related GHGEs by 18,537 ktCO2e, raise £3.4 billion annually, and reduce non-communicable disease mortality through delaying or averting 1,249 UK deaths annually (0.2 % of all UK deaths). By also subsidising foods low in GHGEs and negating the regressive nature of a sales tax, 2,023 deaths (0.4 % of all deaths) could be delayed or averted and 16,453 ktCO2e fewer GHGEs produced.

### Strengths and limitations

This study has several strengths. It is the first study to investigate the potential conflicts between low carbon diets and adverse health consequences through simulating a tax based on GHGEs alongside a tax on SSBs. The tax scenarios analysed and the reported outcomes are the result of this unique price structure and account for resulting substitution and complementing effects. They are therefore not simply equivalent to adding results of previous analyses of a GHGE tax and SSB tax [7, 36]. Our analysis identifies other possible negative health consequences of a GHGE tax despite incorporating an SSB tax. These negative consequences include an increased consumption of *cakes, buns, pastries, and biscuits*, and undesirable changes to individual nutrients such as higher salt and lower vitamin A intake. Furthermore, we simulate realistic dietary scenarios with marginal shifts in consumption of different food groups whilst maintaining an adequate nutritional composition of the overall diet. We use contemporary data from routine sources and simulate not only own-price but also cross-price effects of changes in price. The econometric modelling uses methods which address censoring and ensure theoretical consistency.

There is no single data source that contains all the information required to conduct the modelling in this study and as such we use a range of data sources. All datasets are either sampled or weighted to be representative of the UK population, however, their strengths, limitations, and sample sizes vary. For example, UK census and mortality data are almost complete for the entire UK population whereas the LCF used for estimating baseline diets is based on a sample of 5,531 households [37]. A strength of this work is that both consumption data and price elasticities are based on the same dataset, unlike other studies in this area [13, 20]. However, fully linked datasets would have enabled estimation of differential mortality outcomes by age and sex. Furthermore, as the LCF is measured at the household level rather than the individual level it does not allow for estimating baseline diet or price elasticities by different age or sex groups; in reality these may vary.

Due to the amount of data available in the LCF, it was not possible to disaggregate foods into more than the 32 groups presented in Table 2. As greater resolution is introduced, there are more zero observations in the data and the uncertainty in the estimation increases. This is demonstrated by the particularly large uncertainty intervals around estimates of the change in consumption of some drinks categories (Table 2, Fig. 2). It is also likely that within each food group there will be variation in the changes to purchases of different foods which are assumed to be the same.

Elasticities are based on marginal weekly variations in prices and as such may not truly represent household’s responses to large changes in price (so-called price shocks) such as a 20 % tax on SSBs or a £1.79/kg increase in the price of beef (roughly equivalent to a 5 % to 45 % price increase depending on cut and quality of beef). Our use of unconditional elasticities allows expenditure to shift between food and drink budgets, but not outside of food budgets. It may be that people choose to spend less on their non-food budgets to make up for increased food prices thereby reducing the potential impact of the tax scenarios modelled.

Our greenhouse gas estimates are based on work by Audsley et al.; these represent the most complete collection of life cycle analyses for food consumed in the UK, including estimates based on whether the food is imported from Europe or elsewhere in the world [38]. However, GHGE estimates are unavailable for some food groups found in the LCF and on the FAOSTAT database. Therefore, as with Briggs et al. [7], it was necessary to assume that emissions for some foods were the same as related food products, and where no specific estimates were available for imported products, they were assumed to have the same emissions as if produced elsewhere in the world.

The social cost of carbon is estimated using all sources of GHGEs (from agriculture and elsewhere) and therefore to truly internalise the cost of GHGEs it would be appropriate to raise the price of all food products rather than just those with above average emissions. However, food is a necessity rather than a luxury and the intention of any GHGE based price change would be to shift populations to a less GHGE intensive diet. Therefore, as with Briggs et al., we only estimated price increases on those foods with above averages, with and without subsidies on food with lower emissions, as this is likely to be a more politically acceptable and less regressive policy rather than raising prices across all foods [7, 31]. Similarly, we simulated revenue-neutral policies where foods and drinks with GHGEs below average were subsidised. Sales taxes are regressive however this can be mitigated through redistributing the revenue generated through food subsidies or other progressive tax benefits [47]. Such redistribution is popular with the general public (as discussed by Cornelsen and Carreido, 2015 [48]).

As with previous work in this area, we assume that 100 % of the tax is passed on to the consumer and that all food is consumed [7, 13, 36]. In reality the pass-on rate may be higher or lower than 100 %, however French data from their soft drinks tax suggests that 100 % is reasonable [49]. All food purchased is assumed to be consumed; food waste is not accounted for within the LCF and it is possible that following a change in price, levels of food wastage will fall. Due to the likely differential effect of this between different food groups with different price changes, simulating this effect is not attempted here.

As discussed in the study methodology and consistent with previous modelling and empirical research, we assumed that liquid calories were non-satiating [36, 43–45]. The change in energy intake for each scenario shown in Table 3 is derived entirely from changes to liquid calories. These changes will vary for consumers with different baseline consumption levels of different drinks categories.

Credible intervals are based on the uncertainty in the coefficients used to compute price elasticities. This stage in the modelling has the most uncertainty and is therefore reported in preference to other areas of uncertainty (such as uncertainty in the parameters describing the relationship between food consumed and chronic disease). We do not include an estimate of the model’s structural uncertainty. The PRIME model is a cross-sectional model and therefore does not include any time component of how long the changes to diet would take to manifest in terms of changes to non-communicable disease mortality. The model instead reports the number of annual deaths that would be delayed or averted in the UK population were the population to have always been consuming the new diet compared to baseline.

Finally, all our estimates for changes to health are based on non-communicable disease mortality attributable to diet. We do not include any estimates of changes to morbidity, nor do we estimate the impact on health of reduced global GHGEs.

### Comparisons with other studies

Using 2011 LCF and FAOSTAT data [37, 39], we estimate the total GHGEs related to food consumed in the UK including land use change (up to the retail distribution centre) to be 220,897 ktCO2e, and each simulated tax scenario reduces these emissions by a similar amount (between 7.4 % and 8.5 %). There is variation in the reductions to emissions between different scenarios (for example, scenarios B and D where foods low in GHGEs are subsidised lead to lower reductions than scenarios A and C), although these differences are not statistically significant. Total GHGE reductions are comparable to those previously reported by Briggs et al., who estimated that a similar tax structure to scenario A could reduce emissions from food in 2010 by 18,683 ktCO2e, 7.5 % of 2010 estimates of total emissions related to agriculture [7].

Briggs et al. allowed food as well as liquid calories to change resulting in very different results in terms of the impact on health. They estimated that a scenario equivalent to scenario A would lead to 7,768 deaths delayed or averted (with a 28 kcal reduction), and a scenario equivalent to scenario B would lead to 2,685 more deaths (with an increase in 21 kcal). The difference between the results for scenarios A and B published by Briggs et al. and those reported here is in part driven by changes to energy intake; when Briggs et al. kept energy intake constant, both scenarios A and B led to population health benefits with 1,207 and 2,536 deaths delayed and averted respectively. These are both larger than the equivalent results we report in this study (171 and 1,545 deaths delayed or averted in scenarios A and B respectively when energy remains constant). This due both to different diets in 2010 and 2011 and different tax structures. Data from LCF and FAOSTAT in 2011 suggest that diets are on average lower in GHGEs than in 2010, with the average emissions per kg food being 3.6 kgCO2e in 2011 compared to 4.1 kgCO2e in 2010. This is due to a combination of updated FAOSTAT datasets, changes to UK food import/export patterns, and changes to UK diets. The lower average emissions per kg mean that the simulated tax structure differs between this study and Briggs et al.’s study. Furthermore, price elasticities differ between the studies due to different LCF datasets being used. The fact that more items are taxed in this study than in Briggs et al. (beer is taxed in this study but is not in Briggs et al.) and the levels of taxation are greater (for example, beef is further from the mean and taxed at £1.79/kg compared to £1.76/kg in Briggs et al.) means that revenue is greater. We estimate that scenario A would generate £3.0billion, compared to £2.0billion in the equivalent scenario in Briggs et al. [7]. Given the differences found between this study and Briggs et al., further work should explore the robustness of modelled GHGE tax scenarios.

In this study we take things one step further than in Briggs et al. and identify that a combined food based GHGE tax along with a 20 % tax on SSBs would lead to significant additional health benefits and generate approximately £400million extra in revenue. The effect on change to GHGEs would be minimal compared to a GHGE tax alone. The tax rates simulated in the tax-neutral scenario B are based on the pre-tax baseline diet. Following the tax it is estimated that there would be an overall net loss in revenue of £540million as people move away from taxed products to those that are subsidised. We estimate that this loss would be largely offset by a 20 % SSB tax, resulting in net loss of just £120million in scenario D.

In a study estimating the effects of an SSB tax using similar methodology, Briggs et al. found that a 20 % tax in the UK could reduce calorie intake in adults by around 4 kcal/day [36]. This is in line with our estimates of a 5 kcal/day reduction in energy intake in scenario C. In scenario D, we estimate that there will be a non-significant 1 kcal/day reduction in energy intake, less than in scenario C due to subsidies in scenario D on *fruit juice* and *milk*, which have significant increases in consumption of 5.7 % and 16.5 % respectively.

The scale of the deaths delayed or averted is less than other studies in this area. However, many of the counterfactual scenarios simulated by other studies are much further from current dietary patterns than those used here. For example, Scarborough et al. simulated three sustainable dietary scenarios based on those proposed by the UK Committee on Climate Change Fourth Carbon Budget [12]. These include replacing 50 % of meat with fruit, vegetables and cereals. Friel et al. also simulated large reductions in meat consumption but without considering what calories may be replaced with [50].

Our results are consistent with work by Biesbroek et al. who used the EPIC-NL cohort to estimate GHGEs and mortality outcomes of 40,011 diets [19]. The authors found no significant increase in hazard ratios for all-cause mortality for those eating diets in the highest versus lowest quintiles of GHGEs. However, Biesbroek et al. went on to simulate the effect of a 35 g reduction in meat consumption (approximately a third of total meat consumption) on health and GHGEs. They estimate that, depending on the substitute, this could lead to between a 0 % (if substituted with potatoes) to 19 % (if substituted with fish) reduction in all-cause mortality, with the effects of substituting to other food groups, such as fruit and vegetables, all falling in between.

Finally, Edjabou and Smed simulated the effect of a GHGE tax on food in Denmark. Similar to our results, they identified that revenue neutral scenarios lead to less of a reduction in emissions than non-revenue neutral scenarios, and that saturated fat consumption decreases. They estimated that a tax rate of £26.90 per tCO2e/kg (2011 prices) would reduce emissions by 4 % to 7.9 % (depending on underlying consumption data used), which is comparable to our results of a 7.5 % to 8.5 % reduction following a £28.60 per tCO2e/kg tax.

## Conclusions

This study is the first work in this area to investigate the possible implications on health, GHGEs, and revenue of internalising the social cost of carbon into food prices alongside a SSB tax explicitly designed to improve health. Our results indicate that such a tax structure may lead to significant health benefits, result in meaningful reductions in GHGEs, and raise revenue. Although some aspects of the diet may be healthier, for example due to reduced sugar and saturated fat intake, unintended increases in unhealthy food (such as cakes and biscuits) and nutrients (such as salt) remain. Future work should focus on developing more sophisticated price structures to optimise a healthy population diet that is low in GHGEs.

## Additional files

Additional file 1: **Tabular data.** Sheet 1 shows the matrix of own- and cross-price elasticities for the 32 food and drink categories and sheet 2 shows indicates whether the elasticities are statistically significant or not (1 indicates statistical significance). Elasticity estimates refer to price rise for item listed in top row with respect to quantity change for those listed in first column. (XLS 53 kb)

Additional file 2: **Tabular data.** This file shows the proportion of the food and drink budget spent on each of the 32 food and drink categories. (XLS 35 kb)
